# Immunotherapy-based salvage therapy in advanced soft tissue sarcoma after first-line chemotherapy: a retrospective single-center analysis with emphasis on undifferentiated pleomorphic sarcoma

**DOI:** 10.3389/fimmu.2026.1918276

**Published:** 2026-08-27

**Authors:** Zhichao Liao, Junbo Huang, Haotian Liu, Chao Zhang, Ting Li, Hongyu Wang, Yuanxin Liu, Sijia Ren, Zhiwu Ren, Haixiao Wu, Ruwei Xing, Sheng Teng, Jun Zhao, Wanyi Xiao, Gengpu Zhang, Yun Yang, Jilong Yang

**Affiliations:** 1 Department of Bone and Soft Tissue Tumors, National Clinical Research Center for Cancer, Tianjin Medical University Cancer Institute and Hospital, Tianjin, China; 2 Tianjin’s Clinical Research Center for Cancer, Tianjin, China; 3 State Key Laboratory of Druggability Evaluation and Systematic Translational Medicine, Tianjin, China

**Keywords:** advanced soft tissue sarcoma, chemoimmunotherapy, Chinese cohort, immunotherapy, undifferentiated pleomorphic sarcoma

## Abstract

**Introduction:**

Soft tissue sarcomas (STS) are rare mesenchymal malignancies with limited treatment options in advanced stages. Anthracycline-based chemotherapy remains the standard of care but yields modest outcomes with significant toxicity, underscoring the need for more effective strategies.

**Methods:**

We retrospectively analyzed 56 patients with advanced STS who received PD-1 inhibitor-based therapy at Tianjin Medical University Cancer Institute and Hospital between January 2021 and January 2026. Treatment regimens included immunotherapy alone or in combination with chemotherapy or targeted therapy. The observed primary endpoints were median progression-free survival (mPFS) and median overall survival (mOS). Survival outcomes were analyzed by Kaplan–Meier analysis; factors associated with prognosis were identified by a Cox proportional hazards regression model. Outcomes were compared with a historical cohort of 53 patients treated with chemotherapy alone.

**Results:**

A total of 56 patients were included, and the median follow-up time in the immunotherapy cohort was 19.2 months (range, 1.32–52.8 months). Patients with UPS had a longer median PFS than those with non-UPS histology (P = 0.045). Patients receiving immunotherapy combined with chemotherapy had longer median PFS (P = 0.047) and median OS (P = 0.046) compared with those receiving immunotherapy without chemotherapy. Multivariable Cox regression identified UPS histology (P = 0.023) and immunotherapy combined with chemotherapy (P = 0.047) as factors associated with PFS in advanced STS. Compared with 53 patients who received chemotherapy alone, the immunotherapy-based comprehensive treatment group had longer median PFS in the overall population (P = 0.023); the pure chemoimmunotherapy group (n = 22) also showed longer PFS (P = 0.014). This association was also observed in the UPS subgroup (P = 0.006). Treatment-related adverse events were predominantly grade 1–2; no grade 4–5 severe adverse events or treatment-related deaths occurred.

**Discussion:**

Immunotherapy-based regimens, particularly chemoimmunotherapy, showed a suggested PFS benefit over conventional chemotherapy in advanced STS, with UPS showing a suggested association with disease control. The safety profile was acceptable. Nevertheless, the limitations inherent to the retrospective design, historical controls, modest sample size, and immature overall survival data warrant cautious interpretation.

## Introduction

As rare tumors, sarcomas are a group of highly heterogeneous malignancies of mesenchymal origin with an overall incidence of approximately 5 per 100,000. They are generally classified into soft tissue sarcomas (STS) and bone sarcomas. The latest (5th edition) WHO Classification of Soft Tissue and Bone Tumours describes more than 150 distinct subtypes, of which approximately 70 are STS, accounting for about 1% of adult malignancies (1). Most soft tissue sarcomas metastasize hematogenously to the liver and lungs, which contributes to the difficulty of achieving a cure (2, 3). The majority of STS occur sporadically and without identifiable causes—a circumstance likely attributable to low statistical power for characterizing risk factors resulting from their low incidence and misclassification of histology. Nevertheless, several associated or predisposing factors have been identified, including genetic predisposition, prior treatments, specific diseases, environmental carcinogens, and trauma (4). Surgery with negative margins (R0 resection) is the cornerstone of treatment for localized STS, whereas the management of advanced disease remains challenging owing to its rarity as well as clinical and biological heterogeneity. For patients with advanced STS, palliative surgical resection is also a preferred option, and anthracycline-based palliative therapy remains the standard first-line treatment (5, 6). In this patient population, median progression-free survival (PFS) ranges from 1.6 to 6.8 months and median overall survival (OS) from 10.7 to 19.7 months, while the toxicity of chemotherapy limits its clinical application (7–11). Therefore, more effective treatment strategies beyond conventional chemotherapy and palliative surgery are urgently needed for patients with advanced STS.

Immune checkpoint inhibitors (ICIs) have achieved breakthroughs in several solid tumors. Sarcomas, however, have traditionally been regarded as “cold tumors” lacking cytotoxic lymphocytes within the tumor core, and were initially considered poorly responsive to immunotherapy (12). As the tumor microenvironment (TME) across different STS subtypes has been further characterized, it has become increasingly clear that, given the high heterogeneity of STS, immune infiltration may vary substantially among subtypes. Meanwhile, cellular-level studies have shown that certain sarcoma subtypes possess relatively high immunogenicity, leading to the hypothesis that some sarcomas may respond to immunotherapy (13). Previous research has demonstrated that bone sarcomas respond poorly to immunotherapy and the overall objective response rate (ORR) for STS is below 15% (14–16). Our previous study showed similar results. In that early analysis, no statistically significant difference was found between bone sarcoma and STS patients in median PFS (P = 0.52) or median OS (P = 0.49). For the overall cohort, the median PFS was 6 months, median OS was 16 months, ORR was 10.8%, and disease control rate (DCR) was 18.9%. Subgroup trends suggested a tendency toward poorer PFS in bone sarcoma patients and a more favorable prognosis in relatively immunotherapy-sensitive STS subtypes, though neither reached statistical significance, warranting further validation with larger sample sizes. The core mechanisms underlying the limited efficacy of ICIs may involve low immunogenicity of bone sarcomas, insufficient immune infiltration within the TME, and activation of immunosuppressive pathways (17, 18), as well as substantial variability in tumor mutational burden (TMB) across subtypes—tumors with higher TMB harbor more neoantigens and exhibit greater immunogenicity (e.g., undifferentiated pleomorphic sarcoma, UPS) (19, 20). Moreover, differential treatment responses have also been observed to potentially correlate with the abundance and maturation status of tertiary lymphoid structures (21, 22). Nevertheless, the role of biomarkers in sarcoma immunotherapy and the relationship between predictive markers and prognosis remain unclear (23). Unlike bone sarcomas, certain STS subtypes exhibit high PD-L1 expression and immune cell infiltration, suggesting potential sensitivity to immunotherapy. Previous clinical studies have also demonstrated better responses to immunotherapy in select subtypes compared with others; this phenomenon has likewise been observed in patients with locally advanced or systemic progression (24–27). However, direct data in other researches comparing the efficacy, prognostic factors, and outcomes of immunotherapy-based comprehensive treatment versus conventional chemotherapy in advanced STS remain insufficient. Currently, only reviews comparing different single-arm studies are available, with some studies suggesting advantages in PFS and OS for immunotherapy-based combination strategies with an acceptable safety profile, whereas others do not support this conclusion—and direct comparative analyses are lacking (28–32). Hence, this study retrospectively analyzed latest five-year clinical data (2021-2026) to explore the efficacy, factors associated with prognosis, and safety of immunotherapy-based comprehensive treatment, and compared these outcomes with those of conventional chemotherapy in advanced STS, aiming to provide evidence for individualized treatment of advanced STS.

## Materials and methods

### Study objectives

The primary endpoint of this study was PFS in advanced STS patients who received immunotherapy. Secondary endpoints included ORR, DCR, OS, and safety. In addition, we sought to identify factors associated with prognosis by statistically analyzing all available clinicopathological variables, with the goal of informing new treatment strategies for patients with advanced sarcoma.

### Patients and methods

**Figure 1:**
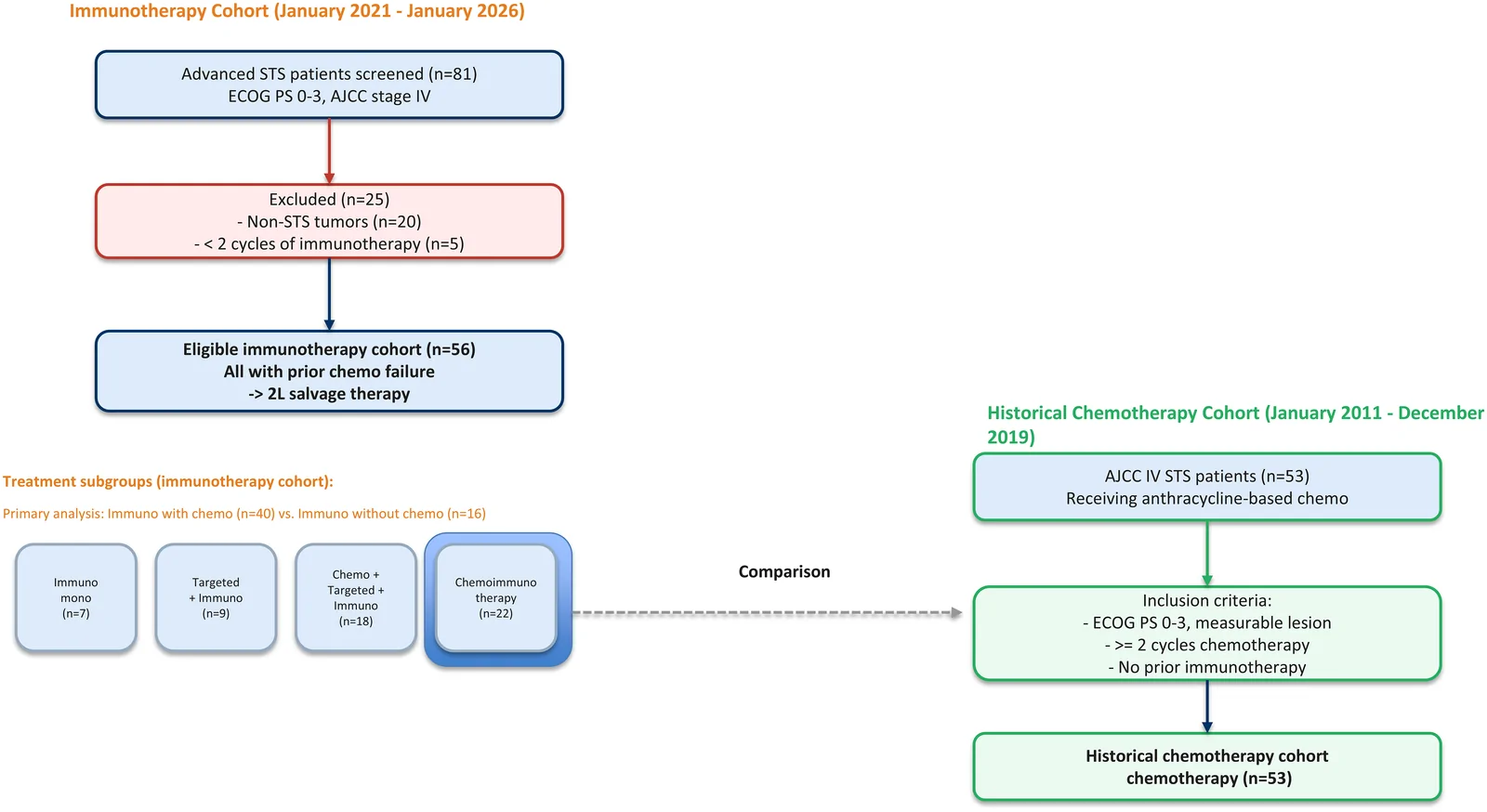
Flowchart of patient enrollment and treatment grouping for advanced soft tissue sarcoma. Immunotherapy cohort (2021–2026): A total of 81 candidate patients were initially screened. After excluding 20 patients with non-STS tumors and 5 patients who received fewer than 2 cycles of immunotherapy, 56 eligible patients with advanced STS were ultimately included. All patients in the immunotherapy cohort had received and progressed on prior first-line chemotherapy. Historical chemotherapy cohort (2011–2019): 53 patients with AJCC stage IV STS who received first-line chemotherapy were identified using comparable eligibility criteria. According to treatment regimen, the immunotherapy cohort was divided into 4 subgroups: immunotherapy monotherapy (n = 7), targeted therapy plus immunotherapy (n = 9), chemotherapy plus targeted therapy plus immunotherapy (n = 18), and pure chemoimmunotherapy (n = 22).

We retrospectively reviewed patients with American Joint Committee on Cancer (AJCC) stage IV soft tissue sarcoma and ECOG performance status scores of 0–3 treated at our center between January 2021 and January 2026, selecting those who had received immunotherapy or immunotherapy-based combination regimens such as chemoimmunotherapy. All patients had pathologically confirmed disease, and each had at least one measurable target lesion for response evaluation. Immunotherapeutic agents included sintilimab, camrelizumab, tislelizumab, pembrolizumab, and toripalimab. Some patients concurrently received conventional chemotherapy, targeted therapy, or both. Chemotherapy regimens included doxorubicin plus ifosfamide (AI) and others; targeted therapy regimens included anlotinib, among others. The historical chemotherapy cohort was assembled from patients with AJCC stage IV soft tissue sarcoma who received first-line anthracycline-based chemotherapy at our center between January 2011 and December 2019. Inclusion criteria were: (1) pathologically confirmed advanced/metastatic STS without prior systemic treatment, (2) ECOG PS 0–3, (3) at least one measurable lesion per RECIST 1.1, (4) at least 2 cycles of chemotherapy received. Patients who had received prior immunotherapy, targeted therapy, or any investigational agent were excluded. Both the immunotherapy cohort and the historical chemotherapy cohort required a minimum of two treatment cycles for inclusion in the efficacy analysis, to ensure adequate treatment exposure for response evaluation. All patients in the immunotherapy cohort had previously received and progressed on first-line anthracycline-based chemotherapy prior to initiating immunotherapy-based treatment as second-line salvage therapy. Based on the specific second-line regimen received, these patients were further categorized into four subgroups: immunotherapy monotherapy (n = 7), targeted therapy plus immunotherapy (n = 9), chemotherapy plus targeted therapy plus immunotherapy (n = 18), and chemoimmunotherapy (n = 22). For the primary analysis, these subgroups were consolidated into two strategies: immunotherapy with chemotherapy (n = 40, combining the chemoimmunotherapy and chemotherapy + targeted therapy + immunotherapy subgroups) and immunotherapy without chemotherapy (n = 16, combining the immunotherapy monotherapy and targeted therapy + immunotherapy subgroups). The choice of regimen in the salvage setting was primarily guided by the patient’s tolerance to prior first-line chemotherapy: patients who had tolerated it well typically received immunotherapy combined with chemotherapy, whereas those with poor tolerance received immunotherapy alone or with targeted therapy, depending on their clinical status. Radiological data, including computed tomography (CT), magnetic resonance imaging (MRI), and B-scan ultrasonography, were reviewed. The collected data were grouped by age, gender, pathological subtype, and treatment regimen for statistical comparison to identify prognostic factors and to assess whether immunotherapy, relative to conventional chemotherapy, offered advantages in efficacy and safety in stage IV STS. Ultimately, we aimed to analyze prognostic factors and identify STS subtypes sensitive to immunotherapy (**Figure 1**).

### Clinical data

Demographic data for all patients included gender and age. Clinical data included diagnosis, primary tumor location, metastatic sites, survival status, date of death, prior treatment history, treatment dates (including drug names and number of cycles), dates of immune checkpoint inhibitor administration, concomitant medications given with ICIs, toxicities reported in patient records and historical laboratory data, ICI discontinuation dates, dates of disease progression, and dates of death. Immune-related toxicities were further evaluated, including the need for intervention. Imaging studies included CT, MRI, or ultrasound to assess the size of target lesions.

### Efficacy evaluation

Tumor responses were rigorously re-evaluated according to Response Evaluation Criteria in Solid Tumors (RECIST) version 1.1. Duration of response was calculated for patients achieving CR, PR, or SD until investigator-assessed progressive disease (PD) or documented intolerable treatment-related adverse events. The primary endpoint was PFS (from treatment initiation to disease progression or death); the secondary endpoints were OS (from treatment initiation to death from any cause), ORR (= CR + PR), and DCR (= CR + PR + SD).

### Safety and toxicity assessment

Safety assessment: All patients were documented and reviewed in detail according to CTCAE version 5.0.

### Statistical analysis

Statistical analysis: SPSS version 26.0 was used for data analysis. Baseline categorical variables were compared between cohorts using the chi-square test or Fisher’s exact test, as appropriate. Survival was assessed by the Kaplan–Meier method with curves drawn accordingly, and the log-rank test was used to identify factors influencing PFS and OS. Median follow-up time was estimated by the reverse Kaplan–Meier method. Multivariable prognostic analysis was performed using a Cox proportional hazards regression model to identify factors associated with PFS. Estimated effects of predictive variables were reported as hazard ratios (HRs) with 95% confidence intervals. A two-sided P < 0.05 was considered statistically significant. Variables were selected for inclusion in the multivariable Cox proportional hazards regression model based on statistical significance in the Kaplan-Meier analysis (P < 0.05) and clinical relevance. UPS histology and immunotherapy combined with chemotherapy met the KM significance threshold (P < 0.05) and were entered as candidate variables. Age, sex, primary tumor site, and combined use of targeted therapy were additionally included as clinically relevant factors that may influence prognosis in soft tissue sarcoma, regardless of their KM P values. No data were missing for the variables included in the final model. Owing to the limited sample size, results from the multivariable analysis are presented as exploratory associations rather than as confirmed factors associated with prognosis. The proportional hazards assumption was tested using Schoenfeld residuals and was satisfied (P > 0.05). The grouping of different PD-1 inhibitors in the primary analysis was a pragmatic analytical decision, as the study was underpowered for agent-specific comparisons. A Kaplan-Meier analysis showed no statistically significant difference in PFS among the five PD-1 inhibitors used (P = 0.988; **Table 1**), although this finding should not be interpreted as demonstrating equivalence given the very small subgroups. Similarly, patients receiving concurrent targeted therapy were included in the immunotherapy-with-chemotherapy or immunotherapy-without-chemotherapy groups based on the rationale that the primary analytical focus was whether chemotherapy was combined with immunotherapy, rather than the presence of targeted agents. Notably, the “pure chemoimmunotherapy” subgroup (n = 22), which excluded patients receiving concurrent targeted therapy, provided an exploratory comparison with the historical chemotherapy group in the UPS subgroup, serving as a sensitivity analysis addressing the potential confounding effect of targeted therapy.

**Table 1:** Results of Kaplan–Meier analysis.

Variable	mPFS (months)	P	mOS (months)	P
Histological subtype		0.045		0.632
UPS	7.984		20.895	
Non-UPS	4.008		25.068	
Primary site		0.220		0.287
Trunk	5.027		20.107	
Extremities	6.045		26.875	
Age		0.550		0.187
< 60 years	6.012		25.068	
≥ 60 years	8.969		20.107	
Gender		0.135		0.215
Male	5.979		22.209	
Female	6.045		23.819	
Combined with chemotherapy		0.047		0.046
Yes	6.965		26.875	
No	3.023		18.661	
Combined with targeted therapy		0.493		0.560
Yes	5.947		22.341	
No	6.965		22.209	
PD-1 inhibitor		0.988		0.560
Camrelizumab	6.045		22.209	
Pembrolizumab	6.045		28.550	
Toripalimab	5.947		23.819	
Tislelizumab	8.969		25.068	
Sintilimab	5.979		18.661	

Red values indicate statistically significant differences (P < 0.05).

The statistical analysis was structured hierarchically. First, within the immunotherapy cohort (n = 56), survival outcomes were compared between the immunotherapy-with-chemotherapy and immunotherapy-without-chemotherapy groups, and between UPS and non-UPS histologies, using Kaplan–Meier analysis and the log-rank test. Second, the historical chemotherapy cohort (n = 53) was introduced as an external reference: survival comparisons were performed between the immunotherapy-based cohort and the chemotherapy cohort, and between the pure chemoimmunotherapy subgroup and the chemotherapy cohort. Subgroup analyses by histological subtype (UPS vs. non-UPS) were prespecified. All comparisons with the historical cohort are presented as exploratory rather than confirmatory, given the inherent differences in treatment period and line of therapy.

### Patient demographics

As of the data cutoff in January 2026, the median follow-up time for the 56-patient immunotherapy cohort, estimated by the reverse Kaplan–Meier method, was 19.2 months (range, 1.32–52.8 months). At cutoff, 33 deaths had occurred in this cohort. A total of 56 patients were enrolled, including 30 males (53.6%) and 26 females (46.4%), with a median age of 56 years (range 18–78 years). The distribution of pathological subtypes was as follows: undifferentiated pleomorphic sarcoma 22 (39.3%), fibrosarcoma 11 (19.6%), synovial sarcoma 5 (8.9%), clear cell sarcoma 4 (7.1%), rhabdomyosarcoma 3 (5.4%), leiomyosarcoma 3 (5.4%), malignant peripheral nerve sheath tumor 2 (3.6%), epithelioid sarcoma 2 (3.6%), angiosarcoma 2 (3.6%), and liposarcoma 2 (3.6%). Treatment regimens were as follows: chemoimmunotherapy 22 (39.3%), chemotherapy combined with targeted therapy and immunotherapy 18 (32.1%), targeted therapy combined with immunotherapy 9 (16.1%), and immunotherapy monotherapy 7 (12.5%). The PD-1 inhibitors administered included toripalimab in 26 (46.4%), pembrolizumab in 18 (32.1%), tislelizumab in 5 (8.9%), camrelizumab in 4 (7.1%), and sintilimab in 3 (5.4%). All patients had an ECOG performance status of 0–3: 35 scored 0 point, 15 scored 1 point, 4 scored 2 points, and 2 scored 3 points (**Table 2**).

**Table 2:** Baseline demographics and clinical characteristics of patients with advanced soft tissue sarcoma.

Characteristic	Chemotherapy (n = 53)	Immunotherapy ± Targeted (n = 16)	Chemoimmunotherapy ± Targeted (n = 40)	P
Gender				0.105
Male	36 (67.9%)	11 (68.8%)	19 (47.5%)	
Female	17 (32.1%)	5 (31.3%)	21 (52.5%)	
Age				0.565
< 60 years	40 (75.5%)	10 (62.5%)	30 (75.0%)	
≥ 60 years	13 (24.5%)	6 (37.5%)	10 (25.0%)	
ECOG PS				0.809
0	33 (62.3%)	8 (50.0%)	27 (67.5%)	
1	16 (30.2%)	5 (31.3%)	10 (25.0%)	
2	2 (3.8%)	2 (12.5%)	2 (5.0%)	
3	2 (3.8%)	1 (6.3%)	1 (2.5%)	
Histological subtype				0.128
UPS	14 (26.4%)	4 (25.0%)	18 (45.0%)	
Non-UPS	39 (73.6%)	12 (75.0%)	22 (55.0%)	

## Results

### Comprehensive chemoimmunotherapy-based treatment shows suggested benefit in STS

The 12-week ORR for the entire cohort was 3.6% (2/56). No patient achieved a complete response (CR). Two patients—one with UPS and one with synovial sarcoma—achieved a partial response (PR). The median PFS for the entire cohort was 6.05 months (**Table 3**). The median follow-up time for the 56-patient immunotherapy cohort was 19.2 months (range, 1.32–52.8 months).

**Table 3:** Efficacy outcomes by treatment and histological subtype.

Group	n	ORR	DCR	mPFS (m)	mOS (m)
Chemotherapy (1L, n = 53)					
Overall	53	2 (3.8%)	27 (50.9%)	4.07	16.66
UPS	14	1 (7.1%)	8 (57.1%)	5.85	10.35
Non-UPS	39	1 (2.6%)	19 (48.7%)	3.12	17.35
Immunotherapy ± Targeted (2L, n = 16)					
Overall	16	0 (0%)	7 (43.8%)	3.02	18.66
UPS	4	0 (0%)	3 (75.0%)	5.98	18.66
Non-UPS	12	0 (0%)	4 (33.3%)	2.04	11.40
Chemoimmunotherapy, all (2L, with or without targeted therapy, n = 40)					
Overall	40	2 (5.0%)	29 (72.5%)	6.97	26.88
UPS	18	1 (5.6%)	15 (83.3%)	7.98	28.35
Non-UPS	22	1 (4.5%)	14 (63.6%)	4.03	25.07
└ Pure chemoimmunotherapy (n = 22)					
Overall	22	1 (4.5%)	16 (72.7%)	6.97	23.82
UPS	10	1 (10.0%)	9 (90.0%)	8.97	17.18
Non-UPS	12	0 (0%)	7 (58.3%)	3.94	25.07

**Figure 2:**
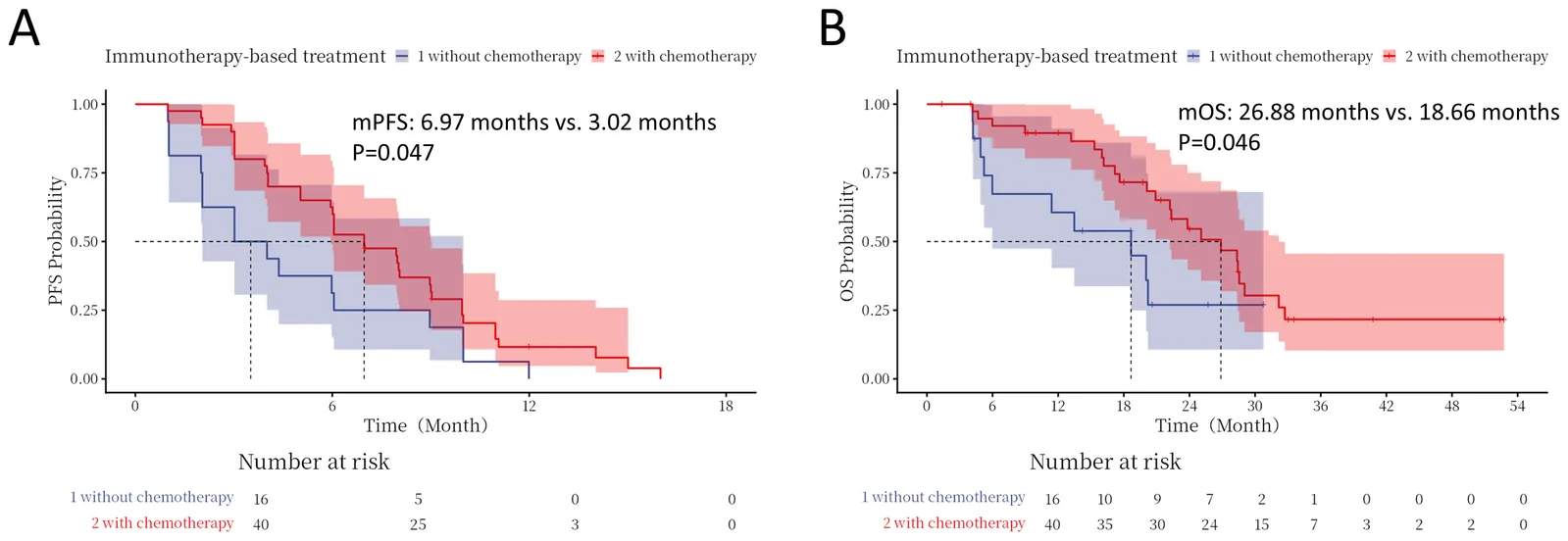
Comparison of PFS and OS between immunotherapy combined with chemotherapy and immunotherapy without chemotherapy. Kaplan–Meier curves for progression-free survival (PFS) in patients with advanced STS stratified by treatment regimen. **(A)**: Median PFS was 6.97 months in the immunotherapy-combined-with-chemotherapy group and 3.02 months in the immunotherapy-without-chemotherapy group; the difference was statistically significant (log-rank test, P = 0.047). **(B)**: Median OS was 26.88 months in the immunotherapy-combined-with-chemotherapy group and 18.66 months in the immunotherapy-without-chemotherapy group; the difference was statistically significant (log-rank test, P = 0.046).

Within the immunotherapy cohort, when treatment regimens were stratified by whether they included chemotherapy, this variable was found to be statistically significant. Patients receiving immunotherapy combined with chemotherapy (i.e., immunotherapy combined with chemotherapy: including both the chemoimmunotherapy group and the chemotherapy + targeted therapy + immunotherapy group) showed longer PFS (mPFS: 6.97 months vs. 3.02 months; P = 0.047) and OS (mOS: 26.88 months vs. 18.66 months; P = 0.046; **Figure 2**).

### UPS histological subtype demonstrated a longer PFS

Initially, Kaplan–Meier analysis was performed across all histological subtypes represented in the cohort; however, owing to the limited sample size within individual subtypes, no statistically significant differences in survival outcomes were observed. When median PFS was compared descriptively across subtypes, patients with UPS exhibited the longest mPFS among all histological categories. This observation is consistent with previous reports suggesting that UPS may be relatively more responsive to immunotherapy than other STS subtypes (19, 20, 38). Accordingly, for subsequent analyses, histological subtypes were dichotomized into UPS and non-UPS groups.

**Figure 3:**
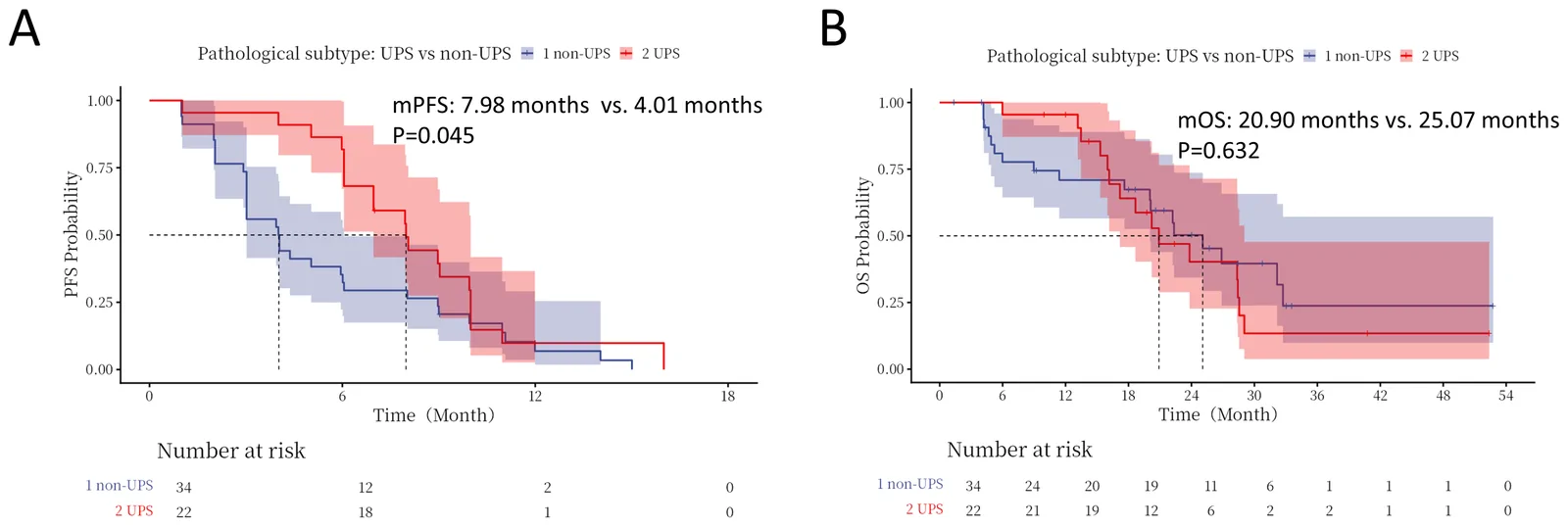
Comparison of PFS and OS between UPS and non-UPS soft tissue sarcoma. Kaplan–Meier curves for progression-free survival (PFS) in patients with advanced STS stratified by histological subtype. **(A)**: Median PFS was 7.98 months for the UPS group and 4.01 months for the non-UPS group; the difference was statistically significant (log-rank test, P = 0.045). **(B)**: No statistically significant difference in overall survival (OS) was observed between the two groups (P = 0.632).

Among the 22 patients with UPS, one achieved PR, and 17 (77.3%) had stable disease (SD) as their best response; of these, 14 progressed within one year. The median PFS for UPS patients was 7.98 months. In Kaplan–Meier analysis, patients with UPS had longer median PFS than those with non-UPS histology (mPFS: 7.98 months vs. 4.01 months; P = 0.045; **Figure 3**). A Kaplan-Meier analysis showed no statistically significant difference in PFS among the five PD-1 inhibitors used in this study (P = 0.988; **Table 1**). This result, together with the limited sample size, justifies pooling different PD-1 inhibitors for the primary analysis as a pragmatic approach. No statistically significant differences were observed with respect to gender, age, other histological subtypes, primary tumor site, or the inclusion of targeted therapy in the immunotherapy combination regimen. Variables that were statistically significant in the univariate analysis (UPS histology, immunotherapy combined with chemotherapy) were entered into a multivariable Cox proportional hazards regression model adjusting for confounders including age and gender. In the multivariable analysis, UPS histology (HR = 0.479, 95% CI: 0.254–0.902, P = 0.023) and immunotherapy combined with chemotherapy (HR = 0.408, 95% CI: 0.168–0.987, P = 0.047) were associated with longer PFS. Given the exploratory nature of this analysis, these associations should be interpreted with caution (**Table 1**, **4**).

**Table 4:** Multivariable Cox proportional hazards model for PFS.

Variable	B	SE	Wald	P	HR	95% CI (lower)	95% CI (upper)
Gender (male vs. female)	−0.119	0.337	0.125	0.724	0.888	0.459	1.717
Immunotherapy combined with chemotherapy vs. immunotherapy without chemotherapy	−0.897	0.451	3.958	0.047	0.408	0.168	0.987
Immunotherapy combined with targeted therapy vs. immunotherapy without targeted therapy	0.287	0.340	0.714	0.398	1.333	0.684	2.596
Primary site			3.755	0.289			
Upper limb	0.682	0.353	3.735	0.053	1.978	0.990	3.952
Head	0.239	0.532	0.202	0.653	1.270	0.448	3.607
Lower limb	0.100	0.677	0.022	0.883	1.105	0.293	4.165
UPS vs. non-UPS	−0.737	0.323	5.190	0.023	0.479	0.254	0.902
Age ≥ 60 vs. < 60 years	0.392	0.246	2.528	0.112	1.480	0.913	2.399

Red values indicate statistically significant differences (P < 0.05).

### Comparison of chemoimmunotherapy and chemotherapy alone in the UPS subgroup

To provide context for the outcomes observed in the immunotherapy cohort, we included data from a historical cohort of patients with advanced STS who received first-line anthracycline-based chemotherapy at our center between January 2011 and December 2019. These patients had metastatic disease at diagnosis without prior systemic treatment. The median follow-up time for this historical chemotherapy cohort, also estimated by the reverse Kaplan–Meier method from the same data cutoff (January 2026), was 16.7 months (range, 1.12–102.14 months); at cutoff, 50 deaths had occurred in this cohort. Baseline characteristics—including age, gender, histological subtype distribution, and primary tumor site—showed no statistically significant differences between the immunotherapy-based comprehensive treatment cohort and the historical chemotherapy cohort, nor between the chemoimmunotherapy subgroup and the historical chemotherapy cohort (all P > 0.05; categorical variables compared by chi-square test or Fisher’s exact test).

### Overall population: immunotherapy-based regimens (including chemoimmunotherapy) versus chemotherapy alone

Overall population: The overall immunotherapy cohort (n = 56) had a longer median PFS (6.05 months vs. 4.07 months; P = 0.023) compared with the chemotherapy group. The pure chemoimmunotherapy group (n = 22) also had a longer median PFS (6.97 months vs. 4.07 months; P = 0.014) compared with the chemotherapy group. However, neither OS for the overall immunotherapy cohort (22.34 months vs. 16.66 months; P = 0.204) nor OS for the pure chemoimmunotherapy group (23.82 months vs. 16.66 months; P = 0.521) showed a statistically significant difference.

### Pure chemoimmunotherapy shows suggested benefit in UPS subtype

**Figure 4:**
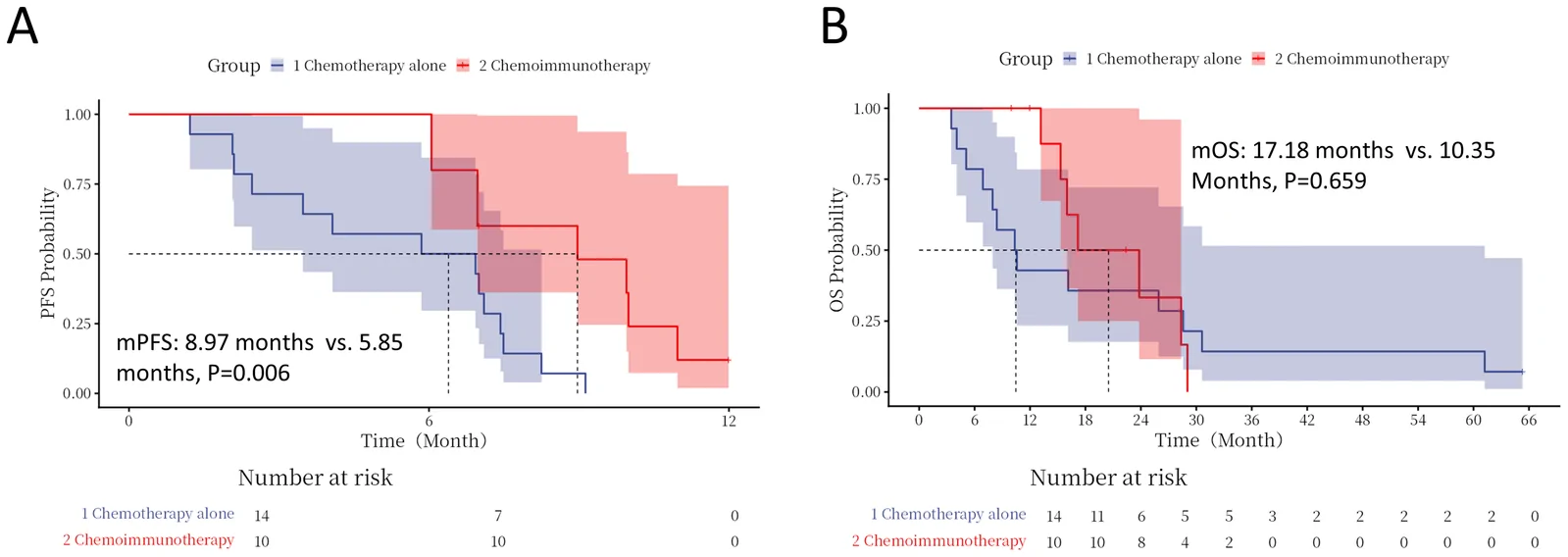
Comparison of pure chemoimmunotherapy versus historical chemotherapy in the UPS subgroup. Kaplan–Meier curves for progression-free survival (PFS) in the undifferentiated pleomorphic sarcoma (UPS) subgroup comparing the pure chemoimmunotherapy and chemotherapy-alone groups. **(A)**: Median PFS was 8.97 months in the chemoimmunotherapy group and 5.85 months in the chemotherapy-alone group, demonstrating benefit from chemoimmunotherapy (log-rank test, P = 0.006). **(B)**: No statistically significant difference in overall survival (OS) was observed between the two groups (P = 0.659).

UPS subtype: The pure chemoimmunotherapy group (n = 10) showed a longer median PFS (8.97 months vs. 5.85 months; P = 0.006) compared with the chemotherapy group, whereas OS (17.18 months vs. 10.35 months; P = 0.659) was not significantly different (**Figure 4**, **Table 5**).

**Table 5:** Comparison of baseline characteristics and efficacy outcomes in patients with UPS across treatment cohorts.

Characteristic	Chemotherapy (1L, n = 14)	Immunotherapy ± Targeted (2L, n = 4)	Chemoimmunotherapy ± Targeted, all (n = 18)	
				Pure chemoimmunotherapy (n = 10)
Age, n (%)				
< 60 years	10 (71.4%)	2 (50.0%)	14 (77.8%)	8 (80.0%)
≥ 60 years	4 (28.6%)	2 (50.0%)	4 (22.2%)	2 (20.0%)
Gender, n (%)				
Male	9 (64.3%)	4 (100%)	6 (33.3%)	4 (40.0%)
Female	5 (35.7%)	0 (0%)	12 (66.7%)	6 (60.0%)
ECOG PS, n (%)				
0	6 (42.9%)	2 (50.0%)	14 (77.8%)	8 (80.0%)
1	7 (50.0%)	2 (50.0%)	4 (22.2%)	2 (20.0%)
2–3	1 (7.1%)	0 (0%)	0 (0%)	0 (0%)
Efficacy				
ORR, n (%)	1 (7.1%)	0 (0%)	1 (5.6%)	1 (10.0%)
DCR, n (%)	8 (57.1%)	3 (75.0%)	15 (83.3%)	9 (90.0%)
mPFS, months	5.85	5.98	7.98	8.97
mOS, months	10.35	18.66	28.35	17.18

Non-UPS subtype: Neither median PFS (3.94 months vs. 3.12 months; P = 0.294) nor median OS (25.07 months vs. 17.35 months; P = 0.462) differed significantly between the pure chemoimmunotherapy group and the chemotherapy group.

### Safety profile and treatment discontinuation

At the time of analysis, 51 patients had discontinued immunotherapy due to disease progression. Eighteen patients (32.1%) experienced immune-related adverse events (irAEs), including immune-related hepatitis in 8 (14.3%), immune-related thyroiditis in 7 (12.5%), immune-related pneumonitis in 3 (5.4%), and skin-related adverse reactions in 3 (5.4%). No grade 4–5 adverse events occurred, nor did any patient discontinue immunotherapy due to adverse events (**Table 6**).

**Table 6:** Treatment-related adverse events (CTCAE version 5.0).

Adverse Event	Grade 1	Grade 2	Grade 3	Grade 4	Total
Gastrointestinal toxicity	28 (50%)	4 (7.1%)	0	0	32 (57.1%)
Hematologic toxicity	12 (21.4%)	8 (14.3%)	1 (1.8%)	0	21 (37.5%)
Immune-related hepatitis	6 (10.7%)	1 (1.8%)	1 (1.8%)	0	8 (14.3%)
Immune-related thyroiditis	7 (12.5%)	0	0	0	7 (12.5%)
Immune-related pneumonitis	2 (3.6%)	1 (1.8%)	0	0	3 (5.4%)
Skin-related adverse reactions	2 (3.6%)	1 (1.8%)	0	0	3 (5.4%)
Immune-related myocarditis	0	0	0	0	0

### PD-1 inhibitor combined with chemotherapy for metastatic undifferentiated pleomorphic sarcoma: a representative case

**Figure 5:**
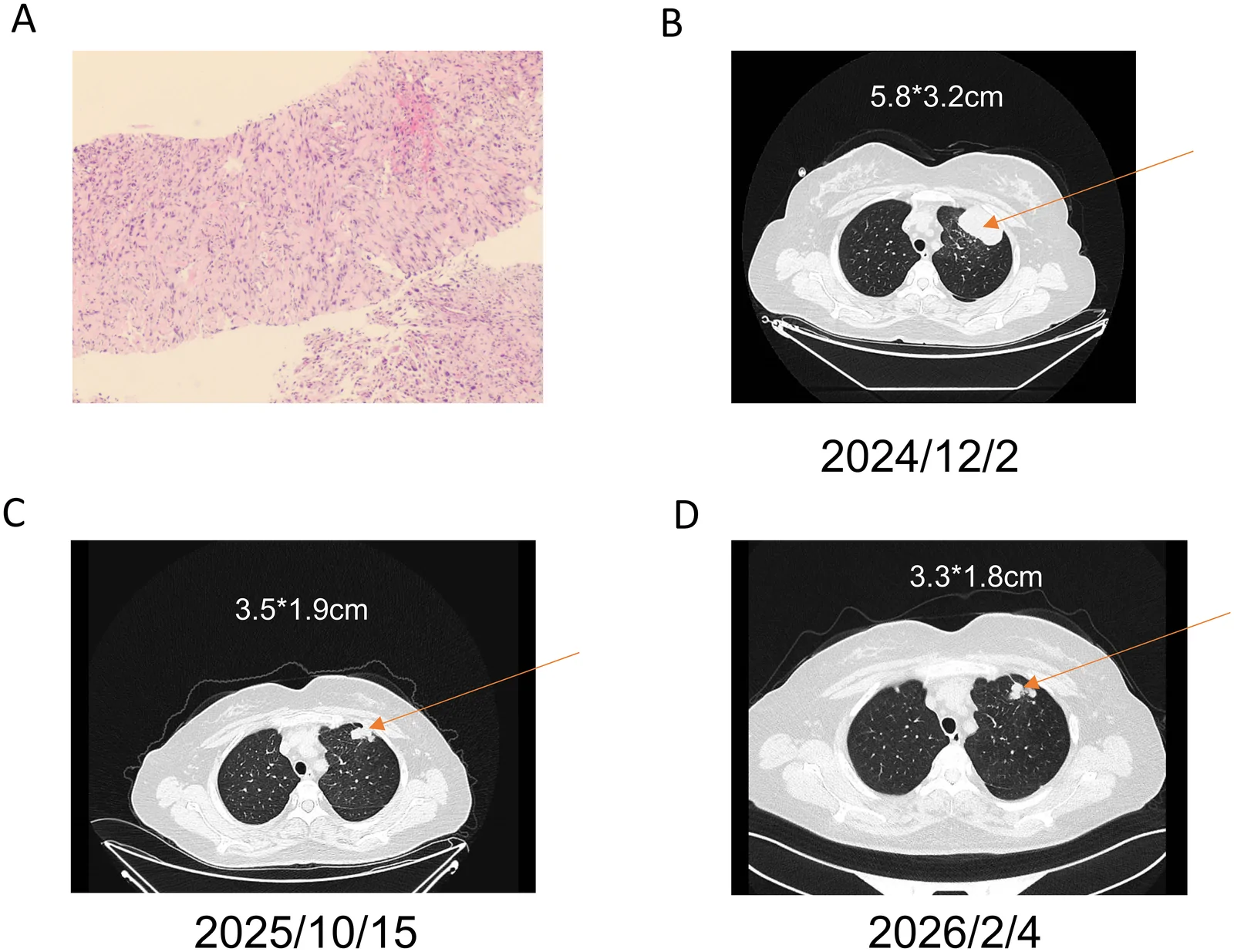
Representative case of PD-1 inhibitor combined with chemotherapy for metastatic undifferentiated pleomorphic sarcoma. Representative images. **(A)**: Needle biopsy of the left lung mass confirmed a pathological diagnosis of metastatic undifferentiated pleomorphic sarcoma. **(B)**: Chest CT before treatment (December 2, 2024) showing a left lung metastasis measuring approximately 5.8 × 3.2 cm. **(C)**: Serial CT scans during treatment (October 15, 2025) showing progressive shrinkage of the left lung metastasis, measuring 3.5 × 1.9 cm. **(D)**: Post-treatment CT (February 4, 2026) showing further reduction of the left lung metastasis to 3.3 × 1.8 cm, with best response confirmed as partial response (PR).

A 44-year-old female with metastatic UPS to the lung received 6 cycles of the AI regimen (ifosfamide 2 g d1–5 + pirarubicin 40 mg d1–2) combined with toripalimab 240 mg every 3 weeks. Serial chest CT scans demonstrated progressive tumor shrinkage from 5.8 × 3.2 cm at baseline to 3.3 × 1.8 cm after 6 cycles, with a best response of partial response. This case illustrates the potential for chemoimmunotherapy to achieve tumor regression in metastatic UPS (**Figure 5**).

## Discussion

The treatment of advanced soft tissue sarcoma (STS) has long faced substantial challenges. For patients with unresectable or metastatic disease, anthracycline-based palliative chemotherapy remains the standard first-line regimen; however, median progression-free survival (PFS) is only 1.6–6.8 months and median overall survival (OS) is 10.7–19.7 months, with the toxicity of chemotherapy limiting its clinical application (7–11). The efficacy of conventional chemotherapy has reached a bottleneck, creating an urgent need for more effective treatment strategies.

In recent years, immune checkpoint inhibitors (ICIs) have achieved breakthroughs in multiple solid tumors. However, early results in the field of sarcoma have been less encouraging. Previous studies have shown that bone sarcomas respond poorly to immunotherapy, with an overall objective response rate (ORR) below 15% for STS (14–16). The core mechanisms are thought to involve low immunogenicity, insufficient immune infiltration in the tumor microenvironment, and activation of immunosuppressive pathways (17, 18). Nonetheless, certain STS subtypes, such as undifferentiated pleomorphic sarcoma (UPS), exhibit relatively high PD-L1 expression and tumor-infiltrating lymphocyte signatures, suggesting the existence of immunotherapy-sensitive subpopulations (25). The SARC028 study and others provided preliminary evidence that pembrolizumab yielded a 40% ORR in UPS (42). A phase II trial showed that pembrolizumab combined with anthracycline-based chemotherapy in patients with unresectable STS demonstrated manageable toxicity and promising activity, with a DCR of 80% and a global ORR of 36.7%; the highest ORR was seen in UPS (4/4 patients) and epithelioid angiosarcoma (1/1 patient), indicating the potential of immunotherapy-based combination strategies (33). More recently, the SU2C-SARC032 randomized trial provided prospective evidence supporting the role of PD-1 inhibition in UPS, demonstrating that the addition of pembrolizumab to preoperative radiotherapy improved outcomes in patients with high-risk extremity soft tissue sarcoma, with consistent benefit observed in the UPS subtype (41). Nevertheless, efficacy in the overall advanced STS population remains limited, and direct evidence is lacking regarding direct comparisons between immunotherapy and conventional chemotherapy, the impact on prognosis of combining immunotherapy with chemotherapy versus not, and factors associated with prognosis, with a particular shortage of real-world data.

Building on previous research, this study retrospectively analyzed five-year clinical data. The baseline characteristics of our sample were similar to those reported in other studies (34–37). Our findings further clarify several key issues. First, this study compared the efficacy of treatment strategies with and without chemotherapy added to immunotherapy, revealing that the combination group (immunotherapy combined with chemotherapy) had longer median PFS (6.97 vs. 3.02 months, P = 0.047) and OS (26.88 vs. 18.66 months, P = 0.046). Multivariable Cox regression identified immunotherapy combined with chemotherapy as a factor associated with PFS (HR = 0.408, 95% CI: 0.168–0.987, P = 0.047). This addresses the gap in the literature regarding comparisons of combination regimens and provides preliminary real-world evidence suggesting a potential synergistic effect between chemotherapy and immunotherapy. The study also found that UPS histology was associated with PFS benefit (mPFS: 7.98 vs. 4.01 months, P = 0.045) and was identified as a factor associated with PFS in the multivariable analysis (HR = 0.479, 95% CI: 0.254–0.902, P = 0.023), suggesting that UPS may derive a greater disease control benefit from immunotherapy-based comprehensive treatment. This is consistent with earlier findings and adds to the existing evidence (38). Moreover, this study found no differences in efficacy across different chemotherapy combination regimens (e.g., AI vs. MAID), which may be attributable to the small sample size and still requires confirmation by prospective randomized controlled studies. Although no grade >3 adverse events were observed in the combination group in our study, the real-world risk of superimposed toxicities from chemotherapy and ICIs should still be carefully monitored.

Second, compared with chemotherapy alone, immunotherapy-based comprehensive treatment was associated with longer PFS in the overall population (6.05 vs. 4.07 months, P = 0.023), and the chemoimmunotherapy group also showed longer PFS compared with the chemotherapy group (6.97 vs. 4.07 months, P = 0.014). This corroborates previous hypotheses and provides preliminary observational data (39, 40). In this exploratory analysis, this study suggests that immunotherapy-based treatment may be associated with longer PFS compared with chemotherapy alone in patients with advanced, unresectable STS, and further demonstrates that this advantage is more pronounced in the UPS subtype (n = 24; 8.97 vs. 5.85 months, P = 0.006), whereas no significant difference was observed in the non-UPS subtypes. This finding extends previous observations by suggesting a histological-subtype-dependent pattern of immunotherapy benefit. In this independent Chinese cohort, disease control following immunotherapy-based treatment was observed in the UPS subgroup, a finding that aligns with the hypothesis that UPS is a relatively immunogenic sarcoma subtype. However, given the modest ORR (4.5%) and the predominant SD rate (77.3%), we characterize this benefit as improved disease control rather than defining UPS as an immunotherapy-sensitive subtype. Notably, the ORR in UPS patients in this study was only 4.5% (1 PR), with no CRs, and the majority achieved SD (77.3%). This contrasts markedly with the 40% ORR reported in the SARC028 study. Possible reasons include: most UPS patients in our study received later-line therapy (most had previously received multiple lines of chemotherapy), whereas SARC028 predominantly included second-line treatment. Nevertheless, achieved SD rates and prolonged PFS carry clinical significance in advanced STS—for patients with chemotherapy-resistant advanced disease, achieving long-term disease stabilization often translates into survival benefit. This observation suggests that, for patients with advanced UPS receiving later-line immunotherapy, the primary benefit may be disease stabilization rather than tumor regression. Furthermore, immunotherapy may yield greater benefit when used in first- or second-line settings than in later lines, a point requiring further investigation.

Third, this study systematically assessed the real-world use of multiple PD-1 inhibitors (sintilimab, camrelizumab, tislelizumab, pembrolizumab, and toripalimab) in STS in a Chinese population and found no significant differences in PFS or OS among these agents.

This study has several limitations that warrant further investigation. First, the retrospective design inevitably carries selection bias; there may be baseline imbalances between the immunotherapy group and the historical control group. Although no statistically significant differences in age, gender, or other baseline characteristics were detected, the historical chemotherapy cohort (2011–2019) and the immunotherapy cohort (2021–2026) were not contemporaneous. This temporal discrepancy may introduce confounding from changes in supportive care, diagnostic modalities, and other period effects; therefore, residual confounding cannot be excluded. Most importantly, the two cohorts differed fundamentally in treatment period (2011–2019 vs. 2021–2026) and treatment line (first-line chemotherapy vs. second-line immunotherapy after first-line failure). The immunotherapy cohort’s status as second-line salvage therapy after chemotherapy failure means these patients carried a worse baseline prognosis, yet they demonstrated longer PFS compared with the first-line chemotherapy cohort—a finding that should be interpreted with caution. These results are hypothesis-generating and require validation in prospective randomized controlled trials. Second, the sample size still needs to be expanded (56 patients in the immunotherapy-based comprehensive treatment), and this is a single-center study, limiting the statistical power for subgroup analyses and multivariable regression, particularly given the very small sample sizes for individual non-UPS subtypes, which preclude separate evaluation. Additionally, although we expanded the baseline comparison to include ECOG PS, certain clinically relevant variables such as detailed comorbidity indices and metastatic burden were not uniformly available across both cohorts, precluding their inclusion. Propensity score matching was considered to reduce confounding, but the fundamental difference in treatment line (first-line vs. second-line) between the cohorts prevents meaningful overlap in propensity score distributions, making PSM infeasible. Future prospective studies with contemporaneous control arms are needed. Third, PD-L1 expression, TMB, MSI status, and TME immune infiltration profiles were not assessed, limiting the ability to mechanistically explain differential ICI sensitivity between UPS and non-UPS subtypes, and precluding exploration of the immunological differences between chemoimmunotherapy, chemotherapy alone, and immunotherapy alone. Fourth, both cohorts applied a per-protocol criterion (≥2 cycles) rather than an intention-to-treat approach, which may introduce selection bias by excluding early progressors. However, this criterion was applied symmetrically to both cohorts to ensure adequate treatment exposure for efficacy evaluation, which is particularly important in a retrospective salvage therapy setting where early treatment discontinuation is common. The exclusion of the 5 patients who received fewer than 2 cycles and the per-protocol approach are acknowledged here as a limitation of the study design.

## Data Availability

The full datasets are not included in the manuscript or supplementary materials to protect patient confidentiality and comply with the regulations of the Medical Ethics Committee of Tianjin Medical University Cancer Institute & Hospital. Only aggregated statistical results and summary tables are presented in the manuscript. Requests to access these datasets should be directed to JY, yangjilong@tjmuch.com.
